# Predicting Six-Month Re-Admission Risk in Heart Failure Patients Using Multiple Machine Learning Methods: A Study Based on the Chinese Heart Failure Population Database

**DOI:** 10.3390/jcm12030870

**Published:** 2023-01-21

**Authors:** Shiyu Chen, Weiwei Hu, Yuhui Yang, Jiaxin Cai, Yaqi Luo, Lingmin Gong, Yemian Li, Aima Si, Yuxiang Zhang, Sitong Liu, Baibing Mi, Leilei Pei, Yaling Zhao, Fangyao Chen

**Affiliations:** 1 Department of Epidemiology and Biostatistics, School of Public Health, Xi’an Jiaotong University Health Science Center, Xi’an 710061, China; 2 Department of Nursing, Xi’an Jiaotong University Health Science Center, Xi’an 710061, China; 3 Department of Radiology, First Affiliate Hospital of Xi’an Jiaotong University, Xi’an 710061, China

**Keywords:** predictive models, six-month re-admission, heart failure, machine learning

## Abstract

Since most patients with heart failure are re-admitted to the hospital, accurately identifying the risk of re-admission of patients with heart failure is important for clinical decision making and management. This study plans to develop an interpretable predictive model based on a Chinese population for predicting six-month re-admission rates in heart failure patients. Research data were obtained from the PhysioNet portal. To ensure robustness, we used three approaches for variable selection. Six different machine learning models were estimated based on selected variables. The ROC curve, prediction accuracy, sensitivity, and specificity were used to evaluate the performance of the established models. In addition, we visualized the optimized model with a nomogram. In all, 2002 patients with heart failure were included in this study. Of these, 773 patients experienced re-admission and a six-month re-admission incidence of 38.61%. Based on evaluation metrics, the logistic regression model performed best in the validation cohort, with an AUC of 0.634 (95%CI: 0.599–0.646) and an accuracy of 0.652. A nomogram was also generated. The established prediction model has good discrimination ability in predicting. Our findings are helpful and could provide useful information for the allocation of healthcare resources and for improving the quality of survival of heart failure patients.

## 1. Introduction

In recent years, with economic growth and population aging, the incidence of cardiovascular disease (CVD) has shown a continuous increase and has become a major public health concern on a global scale [1,2,3]. Heart failure (HF), either preserved or declining left ventricular function, is a serious cardiovascular epidemic of the 21st century [4]. Studies have shown that there were approximately 64.3 million people with HF worldwide in 2017, with a prevalence of 1–2% of adults in developed countries [5,6]. In recent years, the prevalence of HF has also shown a remarkable increase in China. It was reported that nearly half of the global increase in HF cases from 1990 to 2017 was in China and India [6]. The China Cardiovascular Health and Disease Report 2020 suggested that the number of people with current CVD in China is approximately 330 million, with the prevalence of HF reaching 1.3%, or approximately 89 billion for adults, an increase of 44% from 2000 [7,8].

In parallel with measures to reduce the incidence of HF by reducing exposure to risk factors, there is also a need to focus on secondary prevention for those already affected; one of the most important issues is to reduce re-admission rates [9]. The data suggest that half of the patients diagnosed with HF will be re-admitted within a year and 20% will be re-admitted twice or more [5]. By reducing re-admission rates, the quality of life of patients can be improved, the physical, psychological, and financial burden on patients and their families could also be reduced, and healthcare resources can be saved.

The factors associated with the re-admission of HF patients are diverse and the key to reducing re-admission rates is to identify the high-risk groups accurately from multiple perspectives. Accurate prediction will help optimize the stratification of patients by doctors and nurses and guide the rational allocation of limited healthcare resources.

Some models have been developed in developed countries such as the USA, UK, and Japan to predict the risk of re-admission in HF patients [10,11,12,13,14,15]. The variables used in these models can be divided into two categories: the first is the use of a single indicator for prediction; the second is prediction using a combination of variables, as in Bradford et al., who think that employment, status as retired or disabled, >1 emergency department visit in the past 90 days, length of stay >5 days during index visit, and a BUN value >45 mg/dL are associated with an increased risk of 30-day re-admission [16]. That is the prediction from biological factors, clinical factors, psychological factors, and environmental factors. 

However, such models have a few disadvantages. First, the predictors of some models lacked representation. For example, McCabe et al. identified a 6 min walk distance (6MWD ) as an independent predictor of 30-day HF re-admission by constructing a logistic regression model [11]. Chi et al. constructed a Cox regression model to show that frailty was an independent risk factor for 90 unplanned re-admissions and deaths in patients with HF. Frailty is a clinical syndrome that is characterized by a decrease in physical function and stress tolerance. It can be quantified by using the Tilburg Frailty Indicator (TFI) [17]. The predictors in these models were relatively single and did not reflect the impact of clinical and laboratory indicators on the outcome. Second, most of the existing re-admission prediction models are based on European or US medical records databases. However, in previous studies, risk factors for HF have been shown to vary widely across the world; the predictive effect and robustness cannot be guaranteed for Asian populations that cross continental borders due to ethnic, genetic, environmental, cultural, and educational differences [18].

In published studies, the statistical methods used in the establishment of these models were mostly based on logistic regression and Cox proportional risk regression models [10,11,12]. In recent years, artificial intelligence (AI) has been rapidly developing in the healthcare field. Machine learning (ML) is an important branch of AI that has shown an increasingly important role in medical research and clinical care with the widespread use of electronic medical health data [13,14,15,19]. ML-based predictive models cover a wide range of clinical, diagnostic, prognostic, genetic analysis, and pharmacokinetic aspects and perform well, with a variety of machine learning algorithms available, such as random forests, decision trees, support-vector machine (SVM), extreme gradient boosting (XGBoost), multilayer perceptron (MLP), artificial neural network (ANN), etc. [13,20,21,22,23].

Given the unsolved issues in existing models predicting HF re-admission and the advantages of machine learning in the medical field, there is an urgent need to construct an effective prediction model for the Chinese population. Therefore, this study aimed to identify key factors for the six-month re-admission of HF patients, develop prediction models by applying machine learning methods, evaluate the predictive effectiveness of each model, and find the optimal strategy for predicting six-month re-admission of HF patients in China and reducing the burden of disease on patients.

## 2. Methods

### 2.1. Sources of Data

The patient-specific, freely accessible database used in this study was obtained from the PhysioNet data portal (https://physionet.org/content/heart-failure-zigong/1.2/, accessed on 22 November 2021) [24]. The medical records-based database (Hospitalized patients with heart failure: integrating electronic healthcare records and external outcome data) includes 2008 HF patients at the Fourth People’s Hospital of Zigong City, Sichuan, China from December 2016 to June 2019 [25]. HF is defined according to the European Society of Cardiology (ESC) criteria. [26] This database was approved by the ethics committee of the Fourth People’s Hospital of Zigong City (Approval number 2020-010). A detailed introduction and description of the establishment of this database have already been published in another article [25]. 

### 2.2. Study Population

**Figure 1:**
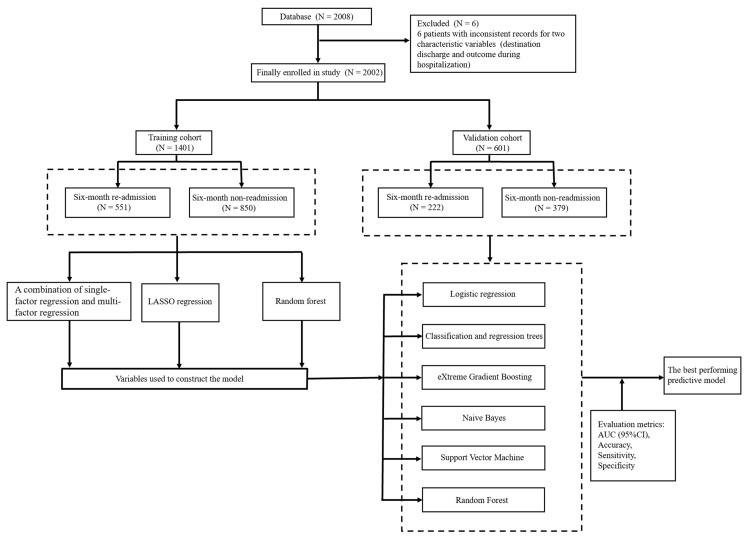
Study design. LASSO regression: least absolute shrinkage and selection operator regression; AUC: area under the curve; CI: confidence interval.

Patients who were diagnosed with HF and had complete data were eligible for inclusion. There were a total of 2008 patients with HF in the database. Six patients had inconsistent records for two characteristic variables (destination discharge and outcome during hospitalization). After exclusion, a total of 2002 patients were included in the study. (Figure 1)

### 2.3. Study Outcome

Based on study implications and the database, the primary outcome of this study was all-cause re-admission to the hospital of HF patients within six months after discharge [11,16,27,28,29]. Study outcomes were obtained by follow-up visits. The electronic medical record will record the outcome of all-cause re-admission and all-cause non-readmission by using telephone follow-up. Unfortunately, the reason for re-admission was not recorded in this retrospective cohort study.

### 2.4. Data Pre-Processing

The data used in this study contained data errors and missing data. For the characteristic variables with a missing rate ≥50%, we were unable to include them in the analysis because the missing rates were too large. The remaining missing variables were filled using the k-nearest neighbor (KNN) method, and K was set to 9. Li et al. used a Monte Carlo method to evaluate the effectiveness of different methods for missing value imputation using the average absolute deviation, average relative deviation, and Type Ⅰ error from the regression analysis as evaluation metrics. Their results show that methods such as KNN imputation, the mean imputation, and the random forest perform better [30]. A total of 2002 study participants with 113 variables were included in the analysis after data management, with six-month re-admission being the target predictor variable.

### 2.5. Variable Selection

A total of 113 features were considered in this study, which included basic patient characteristics information such as age, sex, height, weight, occupation, admission department, visit times, etc. Baseline clinical characteristics such as respiratory rate, systolic blood pressure, diastolic blood pressure, hemoglobin, red blood cells, platelet count, D dimer, the Charlson Comorbidity Index, etc. It also included comorbidities such as diabetes, dementia, liver disease, chronic obstructive pulmonary disease, etc. The baseline characteristics of eligible participants are shown in Supplementary Materials (Table S1).

To capture a wider range of variables associated with six-month re-admission outcomes and to ensure that the variables included in the prediction model were representative and generalizable, we used three different methods for variable screening in the training cohort. (1) Fitting a single-factor logistic regression model for each characteristic variable and the outcome event, correcting for *p*-values using the False Discovery Rate (FDR) method. The FDR is designed to adjust the false positive rate, which keeps the proportion of false positives and true positives within a certain range, allowing us to screen for variables that are truly meaningful for the outcome. Then, fitting a multi-factor logistic regression model fit, with the variables included in the multi-factor model as meaningful characteristic variables; (2) The least absolute shrinkage and selection operator regression (LASSO regression) for high-dimensional variable screening and feature selection. LASSO optimizes the coefficients of the regression by adding a penalty term to the standard multiple regression [31]; (3) Random forest method for screening the importance of the outcome event for variables. Random forest is an ensemble learning method that uses bootstrap resampling to construct different trees and combines all the results to form a decision. [32]

### 2.6. Statistical Analysis

We performed descriptive analysis on all patients included in the study. It described continuous variables conforming to a normal distribution using mean ± standard deviation (SD). Continuous variables with skewed distribution were statistically described using median (interquartile range), and categorical variables were described using frequency (composition ratio). Missing data were imputed by the KNN method using the “DMwR2” package [33] in R.

We randomly divided the dataset into a training set and a test set with a sample size ratio equal to 7:3. The training set was used for variable selection and model construction, while the test set was used for validation.

Six methods including the logistic regression (LR), classification and regression tree (CART), extreme gradient boosting (XGBoost), naive Bayes (NB), support vector machine (SVM), and random forest (RF) were used to develop models. Then, the accuracy, sensitivity, specificity, and the area under the curve (AUC), which is the area under the receiver operating characteristic curve (ROC), were estimated and compared to evaluate the performance of the models and find the best model for predicting the six-month re-admission of HF patients. [34] Finally, nomograms were constructed to improve the interpretability of the models.

All statistical analyses were carried out using the R programming language (version 4.1.2, The R Foundation, Vienna, Austria) and the RStudio software (version 2021.9.1.372, RStudio, PBC, Boston, MA), involving the “DMwR2” [33], “rpart” [35], “randomForest” [36], “xgboost” [37], “pROC” [38] and “e1071” [39] packages. The level of statistical significance was 0.05 (two-tailed).

## 3. Results

### 3.1. Baseline Characteristics

The study included 2002 study subjects and 113 variables covering basic personal information, laboratory indicators, and comorbidities. Of the 2002 patients included in the study, 773 patients had all-cause re-admission within six months and 1229 patients had all-cause non-readmission within six months, giving a six-month all-cause re-admission rate of 38.61% (Table S1). The training cohort had a combined total of 1401 individuals, 551 of whom experienced re-admission, for a six-month all-cause re-admission rate of 39.33%, and the validation cohort had a total of 601 individuals, 222 of whom experienced re-admission, for a six-month all-cause re-admission rate of 36.94%. (Figure 1).

The majority of the included participants were in the age group of 60 to 89 years, accounting for 67.68%. There was a small difference in the percentage of men and women in the study population, with 42% of men and 58% of women. The majority of them were urban residents. Of the total study participants, 93% had a previous admission before this one. The number of patients with whole HF was 1476, followed by 475 with left HF and 51 with right HF. Congestive HF was the most common comorbidity, affecting 1866 of the 2002 HF patients, or 93.21%. Diabetes and moderate to severe chronic kidney disease were the next most common comorbidities, accounting for 23.18% and 23.53% respectively.

### 3.2. Variable selection

**Figure 2:**
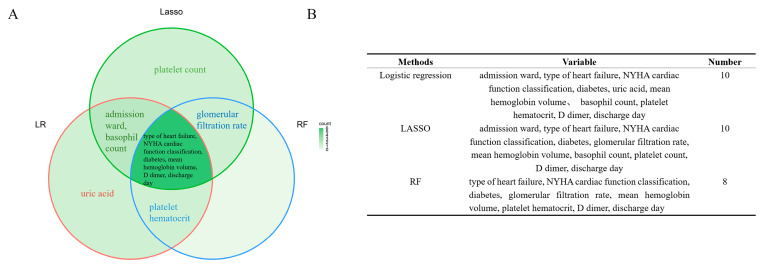
The results of three variable screening methods. RF = random forest; LR = logistic regression; LASSO = least absolute shrinkage and selection operator regression. (**A**): Venn presents the result of the variable selection. (**B**): A total of 10 variables were screened by logistic regression and LASSO regression, and eight variables were screened by random forest, six of which were common to the results of the three methods.

The results of the screening by the three methods are shown below (Figure 2, Figures S1–S4). Combining the results of the three approaches, we consider the following 12 variables: admission ward, type of heart failure, NYHA cardiac function classification, diabetes, uric acid, mean hemoglobin volume, glomerular filtration rate, platelet count, basophil count, platelet hematocrit, D dimer, and discharge day as predictor variables strongly associated with outcome for modeling analysis.

### 3.3. Model Development and Validation

Three different methods were used for variable screening and a total of 12 variables were associated with prognostic outcomes in HF patients and included in model development. (The statistical parameters of the three variable methods are shown in Table S2. The results of the multi-factor logistic regression with 12 variables are shown in Table S3). Six machine learning models were constructed to predict the risk of six-month re-admission in HF patients. (The statistical parameters of the six methods are shown in Table S4). Table S5 shows the model prediction results based on each of the three screening methods. We compared the predictive power of the 10-variable LR model with the 12-variable LR model (Table S6 and Figure S5). Both models performed relatively well, but there were variables in the 12-variable LR model that were not statistically significant. 

**Figure 3:**
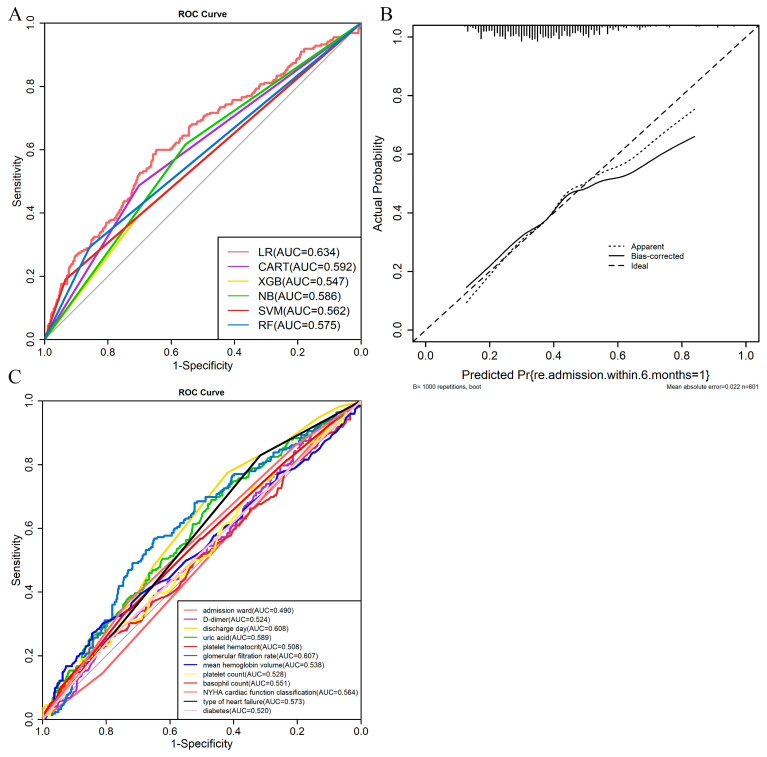
(**A**): The AUC of the 6 machine learning algorithms. (**B**): The calibration curve of the logistic regression model. The ideal line indicates that the model prediction is exactly the same as the actual situation, which is the ideal situation. Apparent and bias-corrected lines indicate the prediction performance of the LR model. Bias-corrected lines. Corrected over-fitting. (**C**): ROC analysis between the 12 clinical variables.

The prediction results of the six machine learning algorithms are shown in Table 1. Comparison of the AUC of six machine learning algorithms is shown in Figure 3A. From the table, we can see that the AUC of the LR model is the highest at 0.634, the remaining five models are worse, and the XGBoost model is the lowest at 0.547. From the perspective of prediction accuracy, the LR, SVM, and RF models are better at over or about 65%, CART is 62.2%, and the lowest is NB, only 57.7%. For the sensitivity and specificity, LR, CART, and NB performed better. Combining the above four evaluation metrics, the LR algorithm is considered to be better for the best prediction, with the CART model being the next best. Using stepwise backward regression, the predictor variables in the final LR model were admission ward, type of heart failure, NYHA cardiac function classification, diabetes, uric acid, mean hemoglobin volume, basophil count, platelet hematocrit, D dimer, discharge day, comprising a list of ten. The calibration plot shows good predictive accuracy between the actual (the ideal line) and predicted probabilities (the apparent and bias-corrected lines) (Figure 3B). The results of the ROC analysis for individual variables are shown in Figure 3C and Table S7. 

### 3.4. Establishment of Nomogram

**Figure 4:**
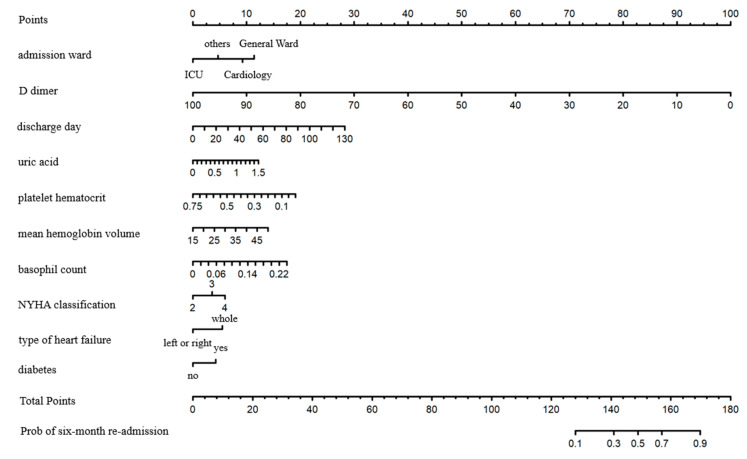
The nomogram of the logistic regression model. NYHA classification: New York Heart classification; ICU: intensive care unit. Points can calculate individual scores for each of the patient’s variables, and the total score is the result of adding the individual scores for each variable. Based on the total score, the probability of all-cause re-admission can be determined.

The final ten variables (admission ward, type of heart failure, NYHA cardiac function classification, diabetes, uric acid, mean hemoglobin volume, basophil count, platelet hematocrit, D dimer, and discharge day) were selected for the logistic regression model. Table 2 describes the included variables by training and validation groups using median (interquartile range) and frequency (composition ratio). In addition, the results of the single-factor regression and the multi-factor regression are shown. The nomogram of the LR model is shown below (Figure 4). The length of the lines in the figure reflects the contribution of each variable entered into the model to the occurrence of a six-month re-admission.

NYHA cardiac function classification, discharge day, uric urea, mean hemoglobin volume, diabetes, and basophil count were associated with higher re-admission risk; when their levels were higher, the patients’ scores were higher. D dimer and platelet hematocrit were associated with lower re-admission. Length of stay in the hospital was also positively associated with the risk of six-month re-admission in HF patients. There was a higher risk of six-month re-admission for patients from the department of cardiology, general ward, and others compared to the intensive care unit (ICU).

## 4. Discussion

Clinical outcomes of HF patients often vary widely depending on the extent of progression and deterioration of the disease. Against the backdrop of global precision prevention and precision treatment, we hope to develop an HF six-month re-admission prediction model that is suitable for the Chinese population, which would undoubtedly be beneficial for both physicians and patients. However, the re-admission of HF patients is influenced by a number of complex factors, of which clinical and biochemical indicators are the most accessible and intuitive to physicians.

In this study, in addition to the incorporation of clinical and biochemical indicators, consciousness, verbal response, mobility, and human factors such as occupation were also considered. This study developed and validated six models to predict the six-month re-admission risk for HF patients based on 12 characteristic variables derived from three variable screening methods. Combining the four evaluation metrics of AUC, accuracy, sensitivity, and specificity, the results showed that the LR model demonstrated superior performance compared to the remaining five models, with an AUC of 0.634, an accuracy of 0.652, a sensitivity of 32.4%, and a specificity of 84.4%.

Different methods have been developed in foreign countries to predict the risk prediction of re-admission in HF patients. For example, Zheng et al. used a state re-admission database to construct a re-admission prediction model with an area under the model curve of 0.59 [12]. Awan et al. constructed an MLP-based approach with a clinical score, logistic regression, random forest decision trees, support-vector machines, and multilayer perception for HF re-admission prediction models, and the results indicated that MLP worked best with 48% sensitivity and 70% specificity [13]. Adler et al. predicted the risk of death in heart failure patients by constructing a boosted decision tree algorithm with an area under the curve (AUC) of 0.88 [14]. Mahajan et al. compared the effectiveness of LR and RF in predicting re-admission using 48 clinical predictors from electronic health record data of 1037 patients with HF from one hospital; their study showed that LR performed better relative to RF with a C-index of 0.65 [40]. Frizzell et al. compared tree-augmented naive Bayesian network, random forest, gradient-boosted three ML methods with traditional logistic regression models for predicting 30-day re-admission in HF patients, and found that traditional regression models showed better predictive performance compared to ML models [29]. This is consistent with the results of this study. Our study concluded that LR was more effective than other models with an AUC of 0.634, a sensitivity of 32.4%, and a specificity of 84.4% by constructing a prediction model with 10 factors containing 2002 patients, which can be considered as a good performance compared with their findings.

It is very difficult to evaluate them with the same criteria because different models are built based on different methods and variables. Most of the ML-based HF re-admission prediction models have not been validated based on an independent prospective cohort of HF patients in China. It is important to note that there are significant differences in diet, ethnicity, and disease prevalence between the Western and Chinese populations. For example, a large number of studies have shown that Chinese populations have a higher incidence of stroke and a lower BMI than Western populations [41,42,43]. The models based on Western populations may not necessarily be applicable to Chinese populations, or even Asian populations, and may even be controversial and misleading.

For variable selection, we used three different methods, incorporating all the variables involved in the modeling analysis, with the plan to capture a wider range of patient characteristics associated with re-admission. Logistic regression, as the traditional and most frequently applied model, provided an intuitive interpretation of the relationship between variables and outcomes but has limitations in identifying variable covariates, whereas the LASSO approach addressed the problem of multiple covariates. The advantages and disadvantages of the three methods complement each other and are more conducive to identifying characteristic variables that are strongly associated with outcome events and the prediction of disease outcomes.

In previous studies, NYHA classification was shown to be a risk factor for 30-day re-admission in patients with HF [44]. Lim et al. used data from the Korean Acute Heart Failure Registry to construct a multivariate logistic regression model to predict 30-day HF re-admission or death, and NYHA classification was included in the final model [10]. In our study, NYHA classification was also a predictor of six-month re-admission for HF, consistent with previous findings. Based on our findings, we recommend that the NYHA classification should be fully considered when studying clinical outcomes in patients with heart failure.

Several studies have shown that serum levels of uric acid are predictive of prognosis in both acute heart failure and chronic heart failure patients [45,46,47]. Uric acid is a purine metabolite. Increased uric acid occurs in HF patients when there is cell death and impaired metabolism. It is thought to be closely associated in clinical practice with the occurrence of adverse cardiovascular events. In our study, we also showed that the risk of six-month re-admission in HF patients increased with increased uric acid levels.

In the study by Davison et al., a multivariate predictive model of 30-day post-discharge all-cause re-admission that included the presence or absence of diabetes had a C-statistic of 0.68, which showed a moderate predictive value. In addition, Davison’s study also suggested that a longer length of stay in the hospital was a critically significant predictor of all-cause re-admission after discharge (though with a *p*-value = 0.0525) [48]. In our study, both diabetes and length of stay were included in the final multivariate prediction model with p-values of 0.0007 and 0.0043, respectively, showing strong significance. Length of stay in the hospital is also positively associated with the risk of six-month re-admission in HF patients, where it cannot be ignored that the length of stay may also imply a more complex physical condition for the patient themselves. Diabetes is a systemic disease and such comorbidities will significantly worsen the short-term prognosis of patients with HF.

The risk of six-month re-admission for patients from the department of cardiology, general ward, and others was higher compared to ICU. Because the majority of patients treated in the ICU have more serious illnesses, they are more likely to have an in-hospital death than patients in other departments.

This study included 2002 HF patients from different age groups, which was a large sample with high statistical validity. In addition, the prediction model based on this database may be more applicable to the Chinese population compared with those established with other populations. Next, different variable screening methods and modeling approaches were used, and the results were highly consistent and reliable. Finally, logistic regression models could be more easily understood (than other models in terms of the relationship between predictor and outcome variables), which increases the potential for wider use by clinicians. The nomogram was highly visual and could provide clinicians with a simple, intuitive, and easy-to-understand tool to identify patients at high risk of six-month re-admission after discharge.

However, there are still some limitations to this study. Firstly, this study is a retrospective modeling study based on a database, which makes it difficult to determine the causal relationship between the selected factors and the re-admission of HF patients. However, we have used different screening and modeling methods and conducted validation to ensure the reliability of the results, to the maximum extent possible. Therefore, the results of this study can still provide reliable quantitative evidence for subsequent prospective studies. Secondly, although the single-center study is not as representative as multi-center studies, many published studies, which are also based on single-center predictive models, have also provided meaningful results for clinical studies. Therefore, our study is also relevant. Thirdly, because there were no electrocardiogram-derived features in the database, they were not considered in our study. However, we extensively explored the significance of the variables present in the database in predicting six-month re-admission. The present study was based on a heart failure database of the Chinese population, which may be of benefit to the Asian population. However, a more generalized model would require a comprehensive database covering a broader population. Furthermore, this study, like most previous studies, did not identify the causes of re-admission [29,49]. However, heart failure occurs mainly in the elderly population, and the all-cause re-admission status is still relevant to improve the quality of patient survival. In addition, we admit that the sensitivity of our model was <50%, but the model had high specificity and good overall accuracy, indicating its good capability of predicting low-risk individuals. The majority of patients who are not re-admitted to the hospital within six months after discharge can also be predicted by the proposed model, which is also of important significance for the reasonable use of healthcare resources. More medical resources can be used for high-risk individuals, thereby reducing their re-admission rates and improving their quality of survival. Finally, there are many modeling approaches based on traditional statistical models and machine learning, but this study does not compare all relevant modeling approaches (e.g., neural networks). However, the six most commonly used modeling approaches were still selected and the results were compared in this study. The results obtained in this study can be considered robust and reasonable.

## 5. Conclusions

As a result, the 10-variable LR model predicting six-month re-admission risk for HF patients established in this study was evaluated by four evaluation metrics indicating that good performance was obtained, and the validation suggested that the results were reasonable. Our findings could provide a reference for clinical care and decision making for HF patients in China and may also provide some value to heart failure re-admission studies in Asian populations.

## Figures and Tables

**Table 1:** The six-month HF re-admission prediction results of the 6 machine learning algorithms.

Model Names	AUC (95%CI)	*p*-Value	Accuracy	Sensitivity (%)	Specificity (%)
LR	0.634 (0.599–0.646)	*p* < 0.001	0.652	32.4	84.4
CART	0.594 (0.486–0.702)	*p* < 0.001	0.622	48.6	70.2
XGBoost	0.547 (0.387–0.707)	*p = 0.019*	0.589	38.7	70.7
NB	0.586 (0.554–0.617)	*p* < 0.001	0.577	61.7	55.4
SVM	0.562 (0.189–0.934)	*p* < 0.001	0.659	18.9	93.4
RF	0.575 (0.293–0.858)	*p* < 0.001	0.649	29.3	85.8

LR = logistic regression; CART = classification and regression trees; XGBoost = extreme gradient boosting; NB= naive Bayes; SVM = support vector machine; RF = random forest.

**Table 2:** Univariate and multivariable logistic regression analyses for six-month heart failure re-admission.

Variables	Re-Admission within 6 Months (Training Cohort)			
	Non-Readmission (N = 850)	Re-Admission (N = 551)	Univariable OR(95% CI, *p*-Value)	Multivariable OR(95% CI, *p*-Value)
Admission ward				
Cardiology	658 (77.4%)	446 (80.9%)		
General Ward	91 (10.7%)	68 (12.3%)	1.10 (0.79–1.54, *p* = 0.570)	1.26 (0.88–1.80, *p* = 0.213)
ICU	7 (0.8%)	1 (0.2%)	0.21 (0.03–1.72, *p* = 0.146)	0.38 (0.05–3.19, *p* = 0.372)
Others	94 (11.1%)	36 (6.5%)	0.57 (0.38–0.85, *p* = 0.005)	0.62 (0.40–0.95, *p* = 0.026)
D dimer/(mg/L)	1.31(0.84,2.27)	1.17(0.81,2.00)	0.91 (0.87–0.96, *p* < 0.001)	0.90 (0.85–0.95, *p* < 0.001)
Discharge day	7 (5,10)	8 (6,11)	1.02 (1.01–1.04, *p* = 0.002)	1.02 (1.01–1.04, *p* = 0.005)
Uric acid (mmol/L)	0.45 (0.36,0.56)	0.47 (0.38,0.60)	2.57 (1.36–4.85, *p* = 0.004)	2.35 (1.18–4.70, *p* = 0.017)
Platelet hematocrit/%	0.17(0.13,0.21)	0.17 (0.13, 0.21)	0.09 (0.02–0.51, *p* = 0.006)	0.07 (0.01–0.44, *p* = 0.004)
Mean hemoglobin volume/(pg)	30.5(28.6,31.8)	30.4(28.5,31.9)	1.05 (1.01–1.08, *p* = 0.006)	1.04 (1.01–1.08, *p* = 0.015)
Basophil count/(×10^9^/L)	0.020(0.020,0.040)	0.030 (0.020, 0.040)	1020.57 (12.03,86551.15, *p* = 0.002)	2127.57 (16.84–268814.95, *p* = 0.002)
NYHA cardiac function classification				
Class 2	172 (20.2%)	72 (13.1%)		
Class 3	443 (52.1%)	286 (51.9%)	1.54 (1.13–2.11, *p* = 0.007)	1.46 (1.06–2.02, *p* = 0.022)
Class 4	235 (27.6%)	193 (35%)	1.96 (1.40–2.74, *p* < 0.001)	1.88 (1.32–2.66, *p* < 0.001)
Type of heart failure				
Left or right	258(30.4%)	110 (20%)		
Whole	592 (69.6%)	441 (80%)	1.75 (1.35–2.25, *p* < 0.001)	1.77 (1.36–2.31, *p* < 0.001)
Diabetes				
No	676 (79.5%)	392 (71.1%)		
Yes	174 (20.5%)	159 (28.9%)	1.58 (1.23–2.02, *p* < 0.001)	1.57 (1.21–2.03, *p* < 0.001)

OR: odds ratio; CI: confidence interval; NYHA cardiac function classification: New York Heart Association cardiac function classification; ICU: intensive care unit.

## Data Availability

The data involved in this study can be obtained at the PhysioNet data portal (https://physionet.org/content/heart-failure-zigong/1.2/, accessed on 22 November 2021).

## Supplementary Materials

The following supporting information can be downloaded at: https://www.mdpi.com/article/10.3390/jcm12030870/s1: Figure S1 (A) Variable selection by LASSO, (B) Plot for LASSO coefficients; Figure S2: The mean out-bag error against different numbers of trees in the forest; Figure S3: Importance scoring of variables by random forest (Mean Decrease Gini); Figure S4: Importance scoring of variables by random forest (Mean Decrease Accuracy); Figure S5: A comparative analysis between ROC with the 12 variables and ROC with the 10 variables; Table S1: Baseline characteristics of all features; Table S2: The criteria or statistical parameters for three variable screening methods; Table S3: The statistical parameters and p-values of the 12 variables; Table S4: The criteria or statistical parameters for the six prediction models; Table S5: The results of the three variable screening methods are used to construct the model separately; Table S6: Comparison of the predictive power of a 10-variable LR model with a 12-variable LR model; Table S7: The results of the ROC analysis for individual clinical variables.
