# Supplementary material for: Predicting Six-Month Re-Admission Risk in Heart Failure Patients Using Multiple Machine Learning Methods: A Study Based on the Chinese Heart Failure Population Database
Source: J Clin Med. 2023 Jan 21;12(3):870. doi: 10.3390/jcm12030870 (PMC9918116; doi:10.3390/jcm12030870)
Supplement: Supplementary file 1 [file jcm-12-00870-s001.zip › jcm-2085132-supplementary.pdf]

Supplementary Materials

Table S1. Baseline characteristic of all features

Variable names	Total(Six-month re-admission)		*p*-value
	non-readmission(N=1229)	re-admission (N=773)	
**destination discharge**			<0.001
dead	9 (0.7%)	0 (0%)	
Healthcare Facility	258 (21%)	180 (23.3%)	
Home	810 (65.9%)	533 (69%)	
Unknown	152 (12.4%)	60 (7.8%)	
**admission ward**			0.014
Cardiology	926 (75.3%)	618 (79.9%)	
General Ward	162 (13.2%)	100 (12.9%)	
ICU	11 (0.9%)	4 (0.5%)	
others	130 (10.6%)	51 (6.6%)	
**admission way**			1
Emergency	583 (47.4%)	367 (47.5%)	
Nonemergency	646 (52.6%)	406 (52.5%)	
**occupation**			<0.001
Famer	156 (12.7%)	57 (7.4%)	
officer	8 (0.7%)	10 (1.3%)	
Urban Resident	1000 (81.4%)	665 (86%)	
worker	14 (1.1%)	3 (0.4%)	
others	51 (4.1%)	38 (4.9%)	
**discharge department**			0.047
Cardiology	1035 (84.2%)	665 (86%)	
General Ward	149 (12.1%)	89 (11.5%)	
ICU	12 (1%)	0 (0%)	
others	33 (2.7%)	19 (2.5%)	
**visit times**			0.012
1	1147 (93.3%)	708 (91.6%)	
2	73 (5.9%)	47 (6.1%)	
3	8 (0.7%)	11 (1.4%)	
4	0 (0%)	6 (0.8%)	
5	1 (0.1%)	1 (0.1%)	
gender			0.363
male	506 (41.2%)	335 (43.3%)	
female	723 (58.8%)	438 (56.7%)	
**body temperature/°C**	36.4(36.2,36.5)	36.3(36.2,36.5)	0.055
**pulse/(beats/min)**	84(71,100)	81(70,96)	0.035
**respiration/(breaths/min)**	19(18,20)	19(18,19)	0.500
**systolic blood pressure/mmHg**	130(118,150)	128(10,144)	<0.001
**Kdiastolic blood pressure/mmHg**	78(68,86)	74(64,84)	0.009
**map/mmHg**	95.33(84,106)	92.67(82,103.33)	<0.001
**weight /Kg**	50(45,60)	50(45,60)	0.299
**height/m**	1.56(1.50,1.62)	1.56(1.50,1.62)	0.792
**BMI/(kg/m^2)**	20.83(18.63,24.44)	20.40(18.37,23.44)	0.098
**type of heart failure**			<0.001
Left or right	378 (30.8%)	148 (19.1%)	
whole	851 (69.2%)	625 (80.9%)	
**NYHA cardiac function classification**			<0.001
2	250 (20.3%)	103 (13.3%)	
3	642 (52.2%)	395 (51.1%)	
4	337 (27.4%)	275 (35.6%)	
**Killip grade**			0.216
1	328 (26.7%)	199 (25.7%)	
2	618 (50.3%)	409 (52.9%)	
3	253 (20.6%)	138 (17.9%)	
4	30 (2.4%)	27 (3.5%)	
**Myocardial infarction**			0.552
0	1138 (92.6%)	722 (93.4%)	

1	91 (7.4%)	51 (6.6%)	
**congestive heart failure**			0.467
0	79 (6.4%)	57 (7.4%)	
1	1150 (93.6%)	716 (92.6%)	
**peripheral vascular disease**			0.852
0	1169 (95.1%)	733 (94.8%)	
1	60 (4.9%)	40 (5.2%)	
**cerebrovascular disease**			0.865
0	1139 (92.7%)	714 (92.4%)	
1	90 (7.3%)	59 (7.6%)	
**dementia**			0.010
0	1172 (95.4%)	715 (92.5%)	
1	57 (4.6%)	58 (7.5%)	
**chronic obstructive pulmonary disease**			0.991
0	1086 (88.4%)	684 (88.5%)	
1	143 (11.6%)	89 (11.5%)	
**connective tissue disease**			0.283
0	1225 (99.7%)	773 (100%)	
1	4 (0.3%)	0 (0%)	
**peptic ulcer disease**			0.201
0	1206 (98.1%)	751 (97.2%)	
1	23 (1.9%)	22 (2.8%)	
**diabetes**			<0.001
0	978 (79.6%)	560 (72.4%)	
1	251 (20.4%)	213 (27.6%)	
**Moderate to severe chronic kidney disease**			<0.001
0	977 (79.5%)	554 (71.7%)	
1	252 (20.5%)	219 (28.3%)	
**hemiplegia**			1
0	1222 (99.4%)	768 (99.4%)	
1	7 (0.6%)	5 (0.6%)	
**solid tumor**			0.853
0	1204 (98%)	759 (98.2%)	
1	25 (2%)	14 (1.8%)	
**liver disease**			0.788
0	1177 (95.8%)	743 (96.1%)	
1	52 (4.2%)	30 (3.9%)	
**AIDS**			0.283
0	1225 (99.7%)	773 (100%)	
1	4 (0.3%)	0 (0%)	
**CCI score**			<0.001
0	31 (2.5%)	25 (3.2%)	
1	501 (40.8%)	269 (34.8%)	
2	439 (35.7%)	262 (33.9%)	
3	211 (17.2%)	156 (20.2%)	
4	41 (3.3%)	51 (6.6%)	
5	6 (0.5%)	9 (1.2%)	
6	0 (0%)	1 (0.1%)	
**type II respiratory failure**			0.411
0	1155 (94%)	734 (95%)	
1	74 (6%)	39 (5%)	
**consciousness**			0.070
clear	1203 (97.9%)	766 (99.1%)	
Responsive to Pain	3 (0.2%)	1 (0.1%)	
Responsive to Sound	13 (1.1%)	6 (0.8%)	
Nonresponsive	10 (0.8%)	0 (0%)	
**Eye-opening**			0.064
1	12 (1%)	1 (0.1%)	
2	3 (0.2%)	0 (0%)	

3	14 (1.1%)	10 (1.3%)	
4	1200 (97.6%)	762 (98.6%)	
**verbal response**			0.072
1	12 (1%)	1 (0.1%)	
2	7 (0.6%)	1 (0.1%)	
3	9 (0.7%)	9 (1.2%)	
4	2 (0.2%)	1 (0.1%)	
5	1199 (97.6%)	761 (98.4%)	
**movement**			0.321
1	17 (1.4%)	4 (0.5%)	
2	1 (0.1%)	0 (0%)	
3	2 (0.2%)	0 (0%)	
4	1 (0.1%)	1 (0.1%)	
5	12 (1%)	10 (1.3%)	
6	1196 (97.3%)	758 (98.1%)	
**respiratory support**			0.422
None	1200 (97.6%)	760 (98.3%)	
IMV	16 (1.3%)	9 (1.2%)	
NIMV	13 (1.1%)	4 (0.5%)	
**oxygen inhalation**			0.417
Ambient Air	63 (5.1%)	47 (6.1%)	
Oxygen Therapy	1166 (94.9%)	726 (93.9%)	
**fio2/%**	33(33,33)	33(33,33)	0.004
**acute renal failure**			0.875
0	1224 (99.6%)	771 (99.7%)	
1	5 (0.4%)	2 (0.3%)1	
**outcome during hospitalization**			<0.001
Alive	1140 (92.8%)	745 (96.4%)	
Dead	10 (0.8%)	0 (0%)	
discharge against Order	79 (6.4%)	28 (3.6%)	
**creatinine enzymatic method/(umol/l)**	82.2(63.1,115.1)	93.9(69.7,131.0)	<0.001
**urea/(mmol/l)**	7.57(5.71,10.92)	8.6(6.24,12.33)	<0.001
**uric acid/(mmol/l)**	0.446(0.353,0.559)	0.477(0.377,0.604)	<0.001
**glomerular filtration rate/(mL/min/1.73 m^2)**	69.78(45.87,93.65)	57.72(39.39,82.29)	<0.001
**cystatin/(mg/L)**	1.48(1.18,2.05)	1.64(1.27,2.29)	<0.001
**white blood cell/(*10^9/L)**	6.52(5.09,8.73)	6.4(5.1,8.35)	0.305
**monocyte ratio/%**	0.07(0.05,0.08)	0.07(0.05,0.08)	0.265
**monocyte count/(*10^9/L)**	0.42(0.32,0.589)	0.42(0.32,0.56)	0.586
**red blood cell/(*10^12/L)**	3.93(3.47,4.35)	3.83(3.40,4.21)	0.001
**coefficient of variation of red blood cell distribution width/%**	14.4(13.5,15.5)	14.4(13.7,15.8)	0.021
**standard deviation of red blood cell distribution width/fL**	47.6(44.9,50.9)	48.1(45.6,52.0)	0.002
**mean corpuscular volume/fL**	93.1(88.9,96.5)	93.5(88.8,97.7)	0.049
**hematocrit/%**	0.362(0.318,0.401)	0.355(0.313,0.391)	0.010
**lymphocyte count/(*10^9/L)**	0.94(0.61,1.29)	0.93(0.66,1.29)	0.259
**mean hemoglobin volume/pg**	30.5(28.7,31.8)	30.7(29,32.2)	0.043
**mean hemoglobin concentration/(g/L)**	326(319,333)	326(318,334)	0.866
**mean platelet volume//fL**	12.2(10.9,13.3)	12.1(10.9,13.1)	0.137
**basophil ratio**	0.004(0.002,0.006)	0.004(0.003,0.006)	<0.001
**basophil count/(*10^9/L)**	0.02(0.02,0.04)	0.03(0.02,0.04)	0.011
**eosinophil ratio**	0.009(0.002.0.022)	0.011(0.003,0.024)	0.027
**eosinophil count/(*10^9/L)**	0.06(0.02,0.13)	0.06(0.02,0.15)	0.022
**hemoglobin/(g/L)**	118(108,133)	116(100,129.5)	0.026
**platelet count/(*10^9/L)**	138(102,178)	132(99,172)	0.062
**platelet distribution width/fL**	16.3(16,16.6)	16.3(16,16.6)	0.272
**platelet hematocrit/%**	0.17(0.13,0.21)	0.16(0.13,0.20)	0.027
**neutrophil ratio**	0.76(0.69,0.83)	0.75(0.67,0.81)	0.012

**neutrophil count/(*10^9/L)**	4.91(3.63,6.98)	4.81(3.54,6.48)	0.144
**D dimer/(mg/l)**	1.28(0.83,2.28)	0.17(0.79,2.02)	0.004
**international normalized ratio**	1.20(1.12,1.34)	1.23(1.13,1.38)	0.002
**activated partial thromboplastin time/s**	33.8(30.6,37.7)	34.6(31.4,38.6)	0.003
**thrombin time/s**	17.2(16.3,17.9)	17.2(16.3,17.9)	0.79
**prothrombin activity/%**	69.5(58.6,78.8)	67.2(53.8,77.4)	0.003
**prothrombin time ratio**	0.21 1.12,1.34(	1.23(1.14,1.38)	0.003
**fibrinogen/(g/L)**	3.04(2.48,3.7)	3.08(2.51,3.69)	0.665
**high sensitivity troponin/(pg/mL)**	0.05(0.02,0.11)	0.063(0.029,0.121)	<0.001
**carbon dioxide binding capacity/ (mmol/L)**	23.9(21.1,26.8)	23.9(20.6,26.6)	0.184
**calcium/ (mmol/L)**	2.28(2.17,2.39)	2.3(2.2,2.41)	0.004
**potassium/ (mmol/L)**	3.85(3.5,4.3)	3.91(3.57,4.39)	0.003
**chloride/ (mmol/L)**	102.8(99,106.1)	102.2(98.2,105.7)	0.032
**sodium/ (mmol/L)**	139.2(136.3,141.7)	138.6(135.2,141.0)	<0.001
**creatine kinase isoenzyme to creatine kinase**	0.17(0.12,0.25)	0.17(0.12,0.26)	0.277
**hydroxybutyrate dehydrogenase to lactate dehydrogenase**	0.83(0.78,0.88)	0.82(0.77,0.86)	0.076
**hydroxybutyrate dehydrogenase/(U/L)**	190(163,227)	188(163,225)	0.393
**glutamic oxaloacetic transaminase /(IU/L)**	26(20,39)	26(20,37)	0.330
**creatine kinase/(IU/L)**	94(66.27,136)	93(66,132)	0.482
**creatine kinase isoenzyme/(IU/L)**	16.25(12.4,21.2)	16.1(12.3,21.2)	0.787
**lactate dehydrogenase/(IU/L)**	229(197,278)	228(196,275)	0.808
**brain natriuretic peptide/(pg/ml)**	729.29(300.4,1685.73)	870.85(319.42,1883.65)	0.11
**nucleotidase/(/U/L)**	3.2(2.3,4.8)	3.22(2.4,4.84)	0.377
**fucosidase/(U/L)**	18.6(15.9,21.7)	18.66(16,22.2)	0.455
**albumin/(g/L)**	36.7(33.4,39.6)	37(33.9,40)	0.069
**white globulin ratio/(U/L)**	1.3(1.1,1.5)	1.4(1.2,1.5)	0.071
**glutamyl transpeptidase/(U/L)**	38.64(22,71)	41(25,74)	0.252
**glutamic pyruvic transaminase/(U/L)**	22(14,37)	20(13,33)	0.010
**indirect bilirubin/(umol/L)**	11.6(7.9,16.7)	12.1(8.00,17.3)	0.172
**alkaline phosphatase(umol/L)**	80(64,101)	79(65,100)	0.815
**globulin/(g/L)**	27.9(24.73,31.4)	27.8(25,30.9)	0.358
**direct bilirubin(umol/L)**	6.4(4.0,10.2)	6.9(4.4,10.6)	0.048
**total bilirubin(umol/L)**	18.1(12.2,27.1)	19.2(12.7,28.3)	0.102
**total bile acid(umol/L)**	5.3(3.1,8.4)	5.17(3.1,8.8)	0.826
**total protein/(g/L)**	64.6(60.2,69.2)	64.99(30.5,69.3)	0.536
**cholesterol/(mmol/L)**	3.6(3.1,4.3)	3.51(2.93,4.17)	<0.001
**low density lipoprotein cholesterol**	1.8(1.4,2.3)	1.74(1.3,2.17)	0.004
**triglyceride/(mmol/L)**	0.99(0.75,1.31)	0.94(0.71,1.31)	0.071
**high density lipoprotein cholesterol/(umol/L)**	1.09(0.88,1.29)	1.07(0.81,1.27)	0.331
**GCS**	15(15,15)	15(15,15)	0.078
**discharge day**	7(5,10)	8(6,11)	<0.001
**age cat**			0.098
(21-29]	1 (0.1%)	3 (0.4%)	
(29-39]	11 (0.9%)	1 (0.1%)	
(39-49]	38 (3.1%)	18 (2.3%)	
(49-59]	66 (5.4%)	40 (5.2%)	
(59-69]	236 (19.2%)	130 (16.8%)	
(69-79]	419 (34.1%)	295 (38.2%)	
(79-89]	399 (32.5%)	244 (31.6%)	
(89-110]	59 (4.8%)	42 (5.4%)	

*The Kruskal-Wallis and x^2 tests were used to compare baseline characteristics of six-month re-admission and six-month non-readmission. The Kruskal-Wallis test was used for non-normal continuous variables and the x^2 test was used for categorical variables.

Table S2. The criteria or statistical parameters for the three variable screening methods

Variable selection methods	The criteria or statistical parameters
a combination of single-factor regression and multi-factor regression was used to screen the study variables	Variables with $p<0.05$ were considered statistically significant. The BH method was used to correct for p values
LASSO regression	$\lambda = 0.02937$
Random forest	N=500

Table S3. The statistical parameters and *p-values* of the 12 variables

Variable	β	*p-value*	Variable	β	*p-value*
admission ward(cardiology)			diabetes(no)		
admission ward(general Ward)	0.2096	0.254	diabetes(yes)	0.4336	0.001
admission ward(ICU)	-0.9988	0.358	platelet count	-0.0007	0.7504
admission ward(others)	-0.4662	0.032	basophil count	7.6110	0.0021
D-dimer	-0.1061	<0.001	NYHA cardiac function classification (class 2)		
discharge day	0.0224	<0.001	NYHA cardiac function classification (class 3)	0.3733	0.0244
uric acid	0.0006	0.1168	NYHA cardiac function classification (class 4)	0.6151	<0.001
platelet hematocrit	-1.9210	0.4202	type of heart failure(left or right)		
glomerular filtration rate	-0.0024	0.2242	type of heart failure(whole)	0.5767	<0.001
mean hemoglobin volume	0.0416	0.0170			

Table S4. The criteria or statistical parameters for the six prediction models

Methods	A combination of single-factor regression and multi-factor regression				LASSO regression				Random forest			
	AUC (95%CI)	Accuracy	Sensitivity	Specificity	AUC (95%CI)	Accuracy	Sensitivity	Specificity	AUC (95%CI)	Accuracy	Sensitivity	Specificity
LR	0.634(0.599,0.646)	0.652	0.324	0.844	0.637(0.568,0.665)	0.667	0.333	0.863	0.634(0.441,0.797)	0.657	0.288	0.973
CART	0.544(0.270,0.818)	0.616	0.270	0.818	0.623(0.486,0.760)	0.659	0.486	0.760	0.573(0.365,0.781)	0.627	0.365	0.781
XGBoost	0.565(0.401,0.728)	0.607	0.401	0.728	0.547(0.419,0.675)	0.581	0.419	0.675	0.556(0.405,0.707)	0.596	0.405	0.707
NB	0.593(0.573,0.613)	0.587	0.613	0.573	0.603(0.562,0.644)	0.592	0.562	0.644	0.559(0.412,0.707)	0.521	0.707	0.412
SVM	0.564(0.203,0.926)	0.659	0.203	0.926	0.563(0.189,0.937)	0.661	0.189	0.937	0.547(0.203,0.892)	0.637	0.203	0.892
RF	0.580(0.356,0.805)	0.639	0.356	0.805	0.578(0.351,0.805)	0.637	0.351	0.805	0.564(0.329,0.799)	0.626	0.329	0.799

Table S5. The results of the three variable screening methods are used to construct the model separately.

prediction models	The criteria or statistical parameters
Logistic regression	Variables with $p<0.05$ were considered statistically significant.
CART	*cp*=0.01, *nsplit*=12
XGBoost	*max_depth*=6, *eta*=0.5
NB	*laplace*=0
SVM	*degree*=3, *cost*=1
RF	N=500

Table S6. Comparison of the predictive power of a 10-variable LR model with a 12-variable LR model.

Model names	AUC (95%CI)	Accuracy	Sensitivity(%)	Specificity(%)
10-variable LR model	0.634(0.599-0.646)	0.652	32.4	84.4
12-variable LR model	0.639(0.554-0.676)	0.657	32.4	85.2

Table S7. The results of the ROC analysis for individual clinical variables.

Variable names	AUC (95%CI)	Accuracy	Sensitivity(%)	Specificity(%)
admission word	0.490(0.106-0.919)	0.631	0	100
D-dimer	0.524(0.142-0.914)	0.631	0	100
discharge day	0.608(0.420,0.775)	0.632	4.50	97.6
uric acid	0.589(0.470,0.689)	0.629	1.80	98.7
platelet hematocrit	0.508(0.126,0.921)	0.631	0	100
mean hemoglobin volume	0.538(0.270,0.844)	0.631	0	100
basophil count	0.551(0.342,0.736)	0.632	3.6	98.15
NYHA cardiac function classification	0.564(0.369,0.731)	0.631	0	100
glomerular filtration rate	0.607(0.568,0.649)	0.631	0	100
platelet count	0.528(0.340,0.730)	0.631	0	100
type of heart failure	0.573(0.317,0.829)	0.631	0	100
diabetes	0.520(0.243,0.797)	0.631	0	100

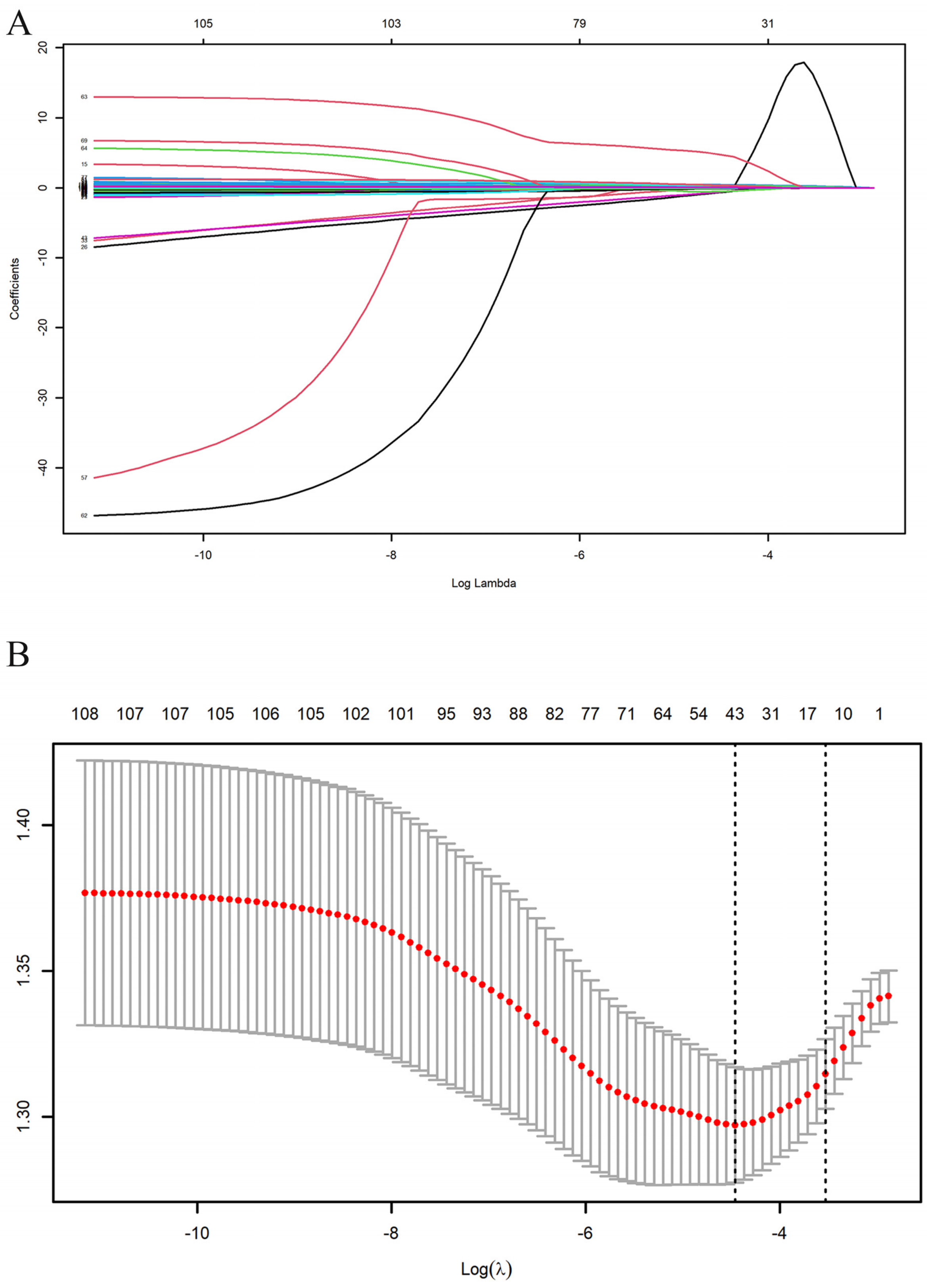

Figure S1. (A)Variable selection by LASSO. As λ increases, the coefficients of some variables keep decreasing until they are zero. (B) Plot for LASSO coefficients. The left dotted line is the value of λ at the smallest mean square error, and the right dotted line is the value of λ one standard error away from the smallest mean square error.
